# Engrailed 1 Mediates Correct Formation of Limb Innervation through Two Distinct Mechanisms

**DOI:** 10.1371/journal.pone.0118505

**Published:** 2015-02-24

**Authors:** Rosa-Eva Huettl, Georg Luxenhofer, Elisa Bianchi, Corinna Haupt, Rajiv Joshi, Alain Prochiantz, Andrea B. Huber

**Affiliations:** 1 Institute of Developmental Genetics, Helmholtz Zentrum München (GmbH)—German Research Center for Environmental Health, Munich, Germany; 2 Institute of Physiology, Ludwig-Maximilians-Universität München, Munich, Germany; 3 Collège de France, Center for Interdisciplinary Research in Biology, Paris, France; Virginia Tech Carilion Research Institute, UNITED STATES

## Abstract

Engrailed-1 (En1) is expressed in the ventral ectoderm of the developing limb where it plays an instructive role in the dorsal-ventral patterning of the forelimb. Besides its well-described role as a transcription factor in regulating gene expression through its DNA-binding domain, En1 may also be secreted to form an extracellular gradient, and directly impact on the formation of the retinotectal map. We show here that absence of En1 causes mispatterning of the forelimb and thus defects in the dorsal-ventral pathfinding choice of motor axons *in vivo*. In addition, En1 but not En2 also has a direct and specific repulsive effect on motor axons of the lateral aspect of the lateral motor column (LMC) but not on medial LMC projections. Moreover, an ectopic dorsal source of En1 pushes lateral LMC axons to the ventral limb *in vivo*. Thus, En1 controls the establishment of limb innervation through two distinct molecular mechanisms.

## Introduction

Execution of coordinated locomotor behaviors relies on precise assembly of circuits between central nervous system neurons and peripheral muscle fibers during embryonic development. Synchronized expression of transcription factor “codes” drives distinct programs of neuronal differentiation along anterior-posterior and medio-lateral axes in the spinal cord, resulting in the formation of molecularly diverse motor neuron subclasses that subsequently innervate different anatomical targets [1]. Neurons innervating limb musculature are clustered in lateral motor columns (LMC) where neurons are organized in patterns that topographically reflect their peripheral targets: motor neurons in the medial aspect (LMCm) project to the ventral limb, while the lateral part (LMCl) consists of neurons innervating dorsal limb musculature [2–4]

The vertebrate limb displays characteristic morphological features distinguishing dorsal from ventral regions, e.g. nails or footpads, respectively. Accordingly, positioning of muscular tissue follows highly organized patterning events. Also within the developing limb, a system of transcription factor codes is utilized for anterior-posterior, proximo-distal, and dorso-ventral patterning: Wnt7a, a secreted factor expressed by the dorsal limb ectoderm, triggers differentiation of dorsal mesenchymal structures by inducing expression of the dorsalizing LIM homeodomain-containing gene *Lmx1b* [5–7].

Expression of the homeodomain transcriptional regulator Engrailed-1 (En1) in ventral limb ectoderm directs development of ventral limb structures [8,9]. Previous studies showed that the functions of limb-patterning signaling pathways are interdependent: mice where either *Lmx1b* or *Wnt7a* were mutated displayed a dorsal-to-ventral conversion of the distal limb bud [5,10]. Mutation of *En1* results in a partial dorsal transformation of the ventral paw and a ventro-proximal expansion of the apical ectodermal ridge (AER; [8]).

Together, these transcriptional networks in limb and spinal cord control expression of guidance cues and their cognate axonal receptors, e.g. members of the ephrin-Eph family, which ultimately determine the specificity of spinal motor connectivity with corresponding limb musculature [11,12]. In chick tectum, Engrailed transcription factors regulate ephrin expression in order to repel Eph-receptor-expressing retinal axons [13,14]. Interestingly, Engrailed, as many homeoproteins, transfers between cells and has additional, non-cell-autonomous functions in gene transcription and local protein translation [15,16]. Engrailed itself plays a direct role in axon guidance events as demonstrated by studies showing that external gradients of soluble Engrailed affect targeting of retinal axon subsets [17].

While the function of En1 in limb patterning and retinotectal targeting is well known, its role during innervation of the limb is only poorly understood. While En1 transcriptionally controls ventralization of the forelimb and hence expression of specific guidance cues, we found that, in addition, soluble En1 specifically repels axons of the lateral but not the medial LMC. Our data therefore establish that En1 critically and directly participates in the dorso-ventral guidance decision of motor nerves innervating the developing forelimb by two distinct mechanisms.

## Experimental Procedures

### Mouse embryo preparation

The genotype of mouse embryos was determined as described for *En1*, where the *LacZ* coding sequence was introduced into the *En1* locus [18] and *Hb9*::*eGFP* [19]. The day of vaginal plug was considered E0.5. Pregnant females were sacrificed by cervical dislocation. E10.5—E12.5 murine embryos were collected in phosphate buffered saline (PBS) or Dulbecco’s Modified Eagle’s Medium (DMEM, Gibco) and decapitated prior to further experimental treatment. Animals were handled and housed according to the German Federal guidelines for the use and care of laboratory animals, and the study was approved by the Helmholtz Zentrum München Institutional Animal Care and Use Committee.

### Immunohistochemistry

The protocol for immunohistochemistry has been described previously [20,21]. The following primary antibodies were used for fluorescent immunohistochemistry on cryosections: rabbit anti-En1 (1:5000), rabbit anti-Lim1 (kindly provided by T.M. Jessell), rabbit anti-EphA4 (1:500, Santa Cruz), rabbit anti-EphA7 (1:100, Santa Cruz), mouse anti-Isl1 39.4D5 (1:50), and mouse anti-Neurofilament 3H5 (1:50, obtained from the Developmental Studies Hybridoma Bank developed under the auspices of the NICHD and maintained by The University of Iowa, Department of Biological Sciences, Iowa City, IA 52242). Antibody staining was visualized using fluorochrome-conjugated secondary antibodies (1:250; Molecular Probes; Jackson Dianova).

### *In situ* hybridization

*In situ* hybridization was performed as described before [20,21] using mouse digoxigenin-labelled probes for *Neuropilin-2* (*Npn-2*, [20]), *Lmx1b* [22], *early B-cell factor 2* (*Ebf-2*, [23]) and *ephrinA5* (a kind gift from Nilima Prakash, [24]).

### Retrograde labeling of neurons

For retrograde labeling of motor neurons, dextran-conjugated Rhodamin (3000MW, Molecular Probes; 6% in PBS + 0,4% TritonX) was injected into either dorsal or ventral musculature of E12.5 mouse embryos. Preparations were incubated for 4 h in aerated D-MEM/F12 medium (Gibco) prior to fixation in 4% Paraformaldehyd (PFA) in PBS for 1 h and cryoprotection in 30% sucrose in PBS overnight [20,21]. To quantitate misprojecting neurons, backfilled Rhodamin-positive neurons were counted, and the percentage of aberrantly projecting neurons was calculated based on immunostaining for Lim1 (LMCl) and Isl1/2 (LMCm). n = 4 for all analyzed paradigms, only embryos with more than 30 retrogradely labeled motor neurons on 12–15 sections of the brachial spinal cord were included into analysis.

### Collapse assay

Dissociated primary motor neurons were prepared from E11.5 *Hb9*::*eGFP* embryos. Spinal cords were extracted and the LMC was excised using a microscalpel under a fluorescent microscope. The tissue was digested at 37°C in 1ml HAM-F10 medium (Gibco) containing trypsin (0.025%, Gibco) and 0.1mg DNAse (Sigma), triturated and centrifuged on a 4% BSA cushion. Cells were resuspended in NeuroBasal medium (Gibco) containing glutamine (200mM, Gibco), glutamate (25mM, Sigma), 0,1% ß-mercaptoethanol (Sigma), 2% horse serum (Invitrogen), 2% B27 supplement (Gibco), 1% penicillin-streptomycin (Sigma), CNTF (10ng/ml, Sigma), BDNF (1ng/ml, Sigma) and GDNF (1ng/ml, Sigma) and plated on glass coverslips coated with poly-L-lysine (10μg/ml, Sigma) and laminin (3μg/ml, Invitrogen). After 5h incubation at 37°C, recombinant Engrailed proteins (En1, En2, En1-SR) or ephrinA2/5 (R&D systems) were added for 1.5–2h. Cultures were fixed in 4% PFA in PBS and processed for immunohistochemical detection of Lim1. Per coverslip, 50 growth cones of each cell population were assessed for collapse by an observer blind to the experimental conditions

### Gain-of-function experiments in chicken

Chick embryos were cultured at 37.5°C until stage 20/21 [25] before injecting ~ 50 nl of En1, En2, En1-SR (20 ng/μl), or PBS into the right dorsal mesenchyme of the developing limb bud using a pulled glass capillary. Injections were repeated every 8 h until embryos reached stage 24/25. Embryos were then fixed in 4% PFA in PBS at 4°C for 1 h and cryoprotected in 30% sucrose overnight. n = 3 for each analyzed condition.

### Analysis of axonal EphA4 distribution

To label LMCl axonal projections in mouse embryos, 20 μm forelimb cryosections were stained for EphA4 and neurofilament and visualized by confocal laser scanning microscopy (Zeiss 510). Dorsal and ventral axon branches at the base of the forelimb were identified using neurofilament staining and the area covered was measured and taken as ROI. Within this ROI, a background substraction was applied in the EphA4 channel and the area covered by EphA4-positive pixels was measured and correlated to the area of neurofilament-positive pixels. To analyze distribution of axonal EphA4 in chicken, 12μm limb cryosections were stained for EphA4 and neurofilament. Average pixel intensities of unsaturated axonal EphA4 stainings were measured with ImageJ software (NIH) and normalized with the average neurofilament pixel intensity of the same area. Ventral and dorsal EphA4 mean intensities for all sections of each limb were then cumulated before calculating the relative EphA4 staining in the dorsal branch compared to total EphA4 signal intensity. n = 3 for mutant and wildtype littermate conditions, 3–6 sections per limb were analyzed.

## Results

### Engrailed 1 in the ventral ectoderm patterns the forelimb

**Fig 1:**
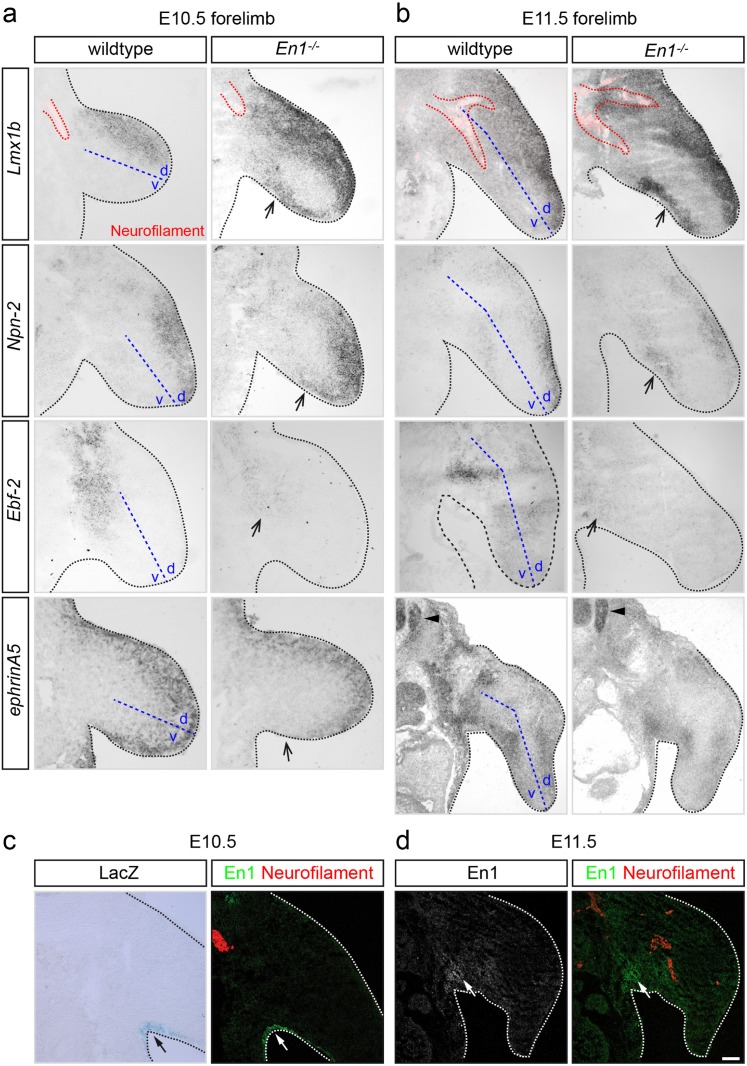
Absence of Engrailed 1 leads to a partial dorsalization of the forelimb. **(a)** *In situ* hybridization against *Lmx1b*, *Npn-2*, *Ebf-2*, and *ephrinA5* on E10.5 coronal sections shows that the dorsally localized genes *Lmx1b* and *Npn-2* extend their expression domain in *En1* ^*-/-*^ mutant mice into ventral areas (arrows), whereas *Ebf-2* and *ephrinA5*, that are usually restricted to the ventral limb mesenchyme in wildtype embryos, show a diminished expression profile (arrows). Neurofilament positive axons converge at the plexus region and have not entered the limb, yet (red dashed line). **(b)** At E11.5, the enlargement of dorsal limb marker domains into ventral areas is maintained in *En1* ^*-/-*^ mutant animals as is the decreased expression of ventral limb markers (arrows). Neurofilament positive axons (red dashed line) have split into dorsal and ventral branches. **(c)** Expression of ß-Galactosidase in *En1* ^*+/-*^ embryos and immunohistochemical stainings for En1 (green) reveal the presence of En1 protein in the ventral ectoderm of the forelimb at E10.5. **(d)** At E11.5, En1 is also found in the ventral forelimb mesenchyme of the axillary region. Scale bar in d equals 100μm for a, b and d and 80μm for c.

Expression of *En1* in the ventral limb ectoderm is essential for patterning, most likely by repressing dorsal differentiation programs induced by Wnt7a [7]. Removal of *En1* results in changes of morphogen expression patterns that are accompanied by evident alterations of the gross dorsal-ventral anatomy. We set out to determine the distribution profile of patterning genes expressed in mesenchyme and guidance cues regulating the dorsal-ventral pathfinding choice in absence of *En1* at distinct developmental time points: at E10.5 motor and sensory axons have formed spinal nerves that have arrived at the plexus region at the base of the limb but not yet entered forelimb tissue (red dashed line, Fig. 1a). At E11.5, axons from spinal nerves contributing to forelimb innervation have been sorted into target specific bundles and have maneuvered this choice point (red dashed line, Fig. 1b).

At E10.5, expression of *Lmx1b* is restricted to the dorsal mesenchyme in wildtype embryos, however, in embryos where *En1* was mutated, an ectopic domain of *Lmx1b* expression was observed in the ventral limb (Fig. 1a). Similarly, the axon guidance receptor *Npn-2* is expressed in the dorsal limb mesenchyme in wildtype embryos, while its dorsal expression domain was enlarged into ventral territories in absence of *En1* (Fig. 1a). While the expression domains of both dorsal mesenchyme markers were broadened into ventral territories, the forelimb was not completely dorsalized in absence of *En1*, as shown by the expression of the ventral mesenchyme marker *Ebf2* [26,27] that is strongly reduced, though not completely abolished in *En1* mutants when compared to wildtype littermates (Fig. 1a).

The repulsive axon guidance cue ephrinA5 is expressed in the ventral limb mesenchyme and governs the dorsal pathfinding of LMCl axons [12,28]. At E10.5, it is expressed in dorsal and ventral limb mesenchyme and the adjacent body wall in wildtype embryos. In *En1* ^*-/-*^ mutant embryos, *ephrinA5* expression is still observed in dorsal limb mesenchyme. In the ventral part of the limb, however, expression of *ephrinA5* is strongly reduced in the regions around the axilla (Fig. 1a).

Similar aberrant expression patterns were also observed at E11.5: in embryos where *En1* was knocked out *Lmx1b* and *Npn-2* were ectopically expressed in ventral limb regions, while *Ebf2* was strongly reduced when compared to wildtype littermates (Fig. 1b). *EphrinA5* showed a patterned expression in wildtype embryos at E11.5 in the dorsal and ventral forelimb. In mutant embryos, patterned expression was still observed, however, expression was diminished in the limb and within the plexus region, while e.g. in the spinal cord and dorsal root ganglia, comparable expression values as in wildtype littermates were observed (Fig. 1b, arrowheads). These findings indicate that the function of En1 in forelimb pattering cannot be compensated for by other cues in absence of *En1* and persists throughout the period of limb innervation.

En1 protein itself is present in ventral ectodermal cells and in the adjacent ventral mesenchyme area by E10.5 as shown by immunohistochemical analyses and staining for ß-Galactosidase in *En1* ^*+/-*^ embryos [18], Fig. 1c). By E11.5, En1 protein has spread in the axillary region and appears to be not localized fully within the nucleus, anymore, which may pinpoint towards an alternate role of En1 during establishment of peripheral projections (Fig. 1d).

Together, these data demonstrate that *En1* governs the tightly controlled and restricted spatial expression of genes that pattern the developing ventral forelimb.

### The dorsal-ventral choice of brachial LMC axons is disturbed in absence of En1

**Fig 2:**
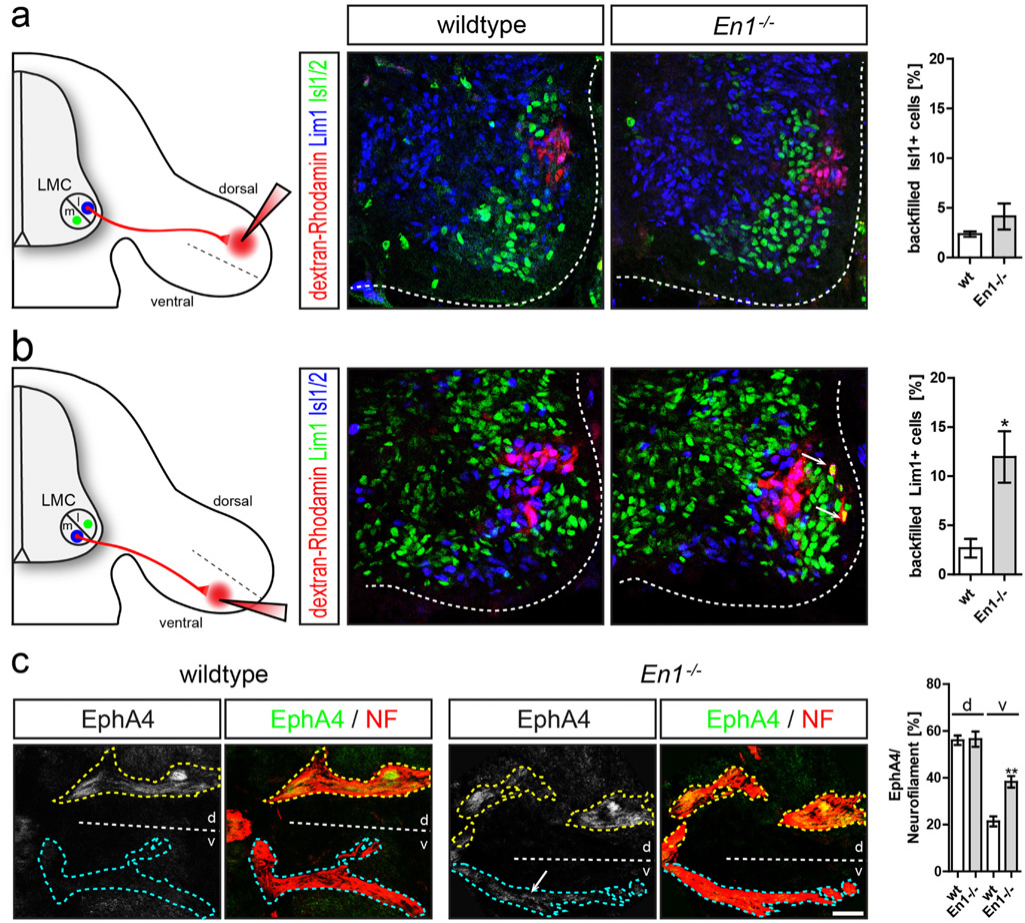
Lateral LMC axons are misguided in absence of En1. **(a)** Retrograde tracing from dorsal forelimb mesenchyme labels motor neurons in the LMCl (Lim1, blue) and showed no significant increase of aberrantly projecting LMCm neurons (Isl1/2-positive, green) in *En1* ^*-/-*^ mutant embryos when compared to wildtype littermates. **(b)** Retrograde tracing from ventral forelimb mesenchyme labels motor neurons in the LMCm (Isl1/2, blue) and revealed a significantly increased number of Lim1-positive LMCl motor neurons that aberrantly project to the ventral limb in *En1* ^*-/-*^ mutant embryos (Lim1-positive, green and Rhodamine-positive, red). **(c)** Staining for neurofilament in E12.5 murine embryos shows bifurcation of limb innervating axons into a dorsal (yellow dashed line) and a ventral (cyan dashed line) branch. In *En1* ^*-/-*^ mutant embryos, a significantly higher proportion of EphA4-positive axons is observed in the ventral branch, while in wildtypes EphA4 is largely restricted to the dorsally projecting branch. Scale bar in c equals 50μm for all panels.

Does the partially dorsalized developing forelimb observed in *En1* mutant embryos impinge on the correct formation of brachial motor trajectories? To investigate the accuracy of the dorsal-ventral pathfinding choice, we retrogradely labeled motor neuron cell bodies by injecting fluorescence-conjugated dextran into either dorsal or ventral forelimb muscles of E12.5 *En1* ^*-/-*^ mutant embryos and wildtype littermates. We then assessed the presence of retrogradely transported dextran in the cell bodies of Lim1-positive LMCl motor neurons that normally only innervate dorsal muscles and Isl1/2-positive LMCm motor neurons that supply ventral limb musculature (Fig. 2).

Retrograde tracing from dorsal musculature in wildtype resulted in primarily Rhodamine-dextran/Lim1 double positive LMCl neurons, with only 2.35% ± 0.26 SEM of dorsally dextran-labeled neurons expressing Isl1/2 (Fig. 2a). In *En1* ^*-/-*^ mutant embryos, retrograde tracing from dorsal limb mesenchyme did not lead to an increase of retrogradely labeled and thus misprojecting Isl1/2-positive motor neurons of the LMCm (4.13% ± 1.3 SEM, p = 0.48).

Quite contrary to these findings, in *En1* ^*-/-*^ mutants significantly more Lim1-positive LMCl motor neurons were labeled by ventral tracer injections (11.95% +/- 2.6 SEM, Fig. 2b) when compared to wildtype littermates (2.67% ± 0.95 SEM, p<0.03) and therefore identified as aberrantly projecting to the ventral limb. Thus, the percentage of misguided lateral LMC axons that choose an inappropriate path to the ventral forelimb was increased 6-fold in the absence of *En1*.

As an independent way to assess the trajectories of LMCl neurons we examined EphA4 expression by LMCl neurons and their axons. In accordance with our observations employing retrograde tracing, and in contrast to the situation in wildtype mice, EphA4 positive axons were not only found in the dorsal limb, but also projected to the ventral half of the forelimb in *En1* ^*-/-*^ mutant embryos (Fig. 2c). Quantitative immunofluorescence analysis of EphA4 expression on axonal projections in the limb at E12.5 revealed a higher level of EphA4 expression on ventral axons in *En1* mutants (38.28% ± 2.47 SEM) when compared to wildtype littermates (21.39% ± 2.16 SEM, p<0.01). EphA4 expression in the dorsal limb mesenchyme, which was found to take part in muscle precursor migration and reverse signaling through axon-bound ephrins [29,30], and which together with EphA7might act as an attractant for specific axons if ectopically expressed in the ventral limb [31–34], was not altered in *En1* ^*-/-*^ mutant embryos (S1 Fig a and b.). These results corroborate our retrograde tracing experiments and demonstrate the presence of aberrantly projecting lateral LMC axons in the ventral limb.

Therefore, these data show that En1 is required for the establishment of accurate medial and lateral LMC projections to the corresponding muscles in the ventral and dorsal forelimb.

### Engrailed 1 specifically repels LMCl axons *in vitro*

**Fig 3:**
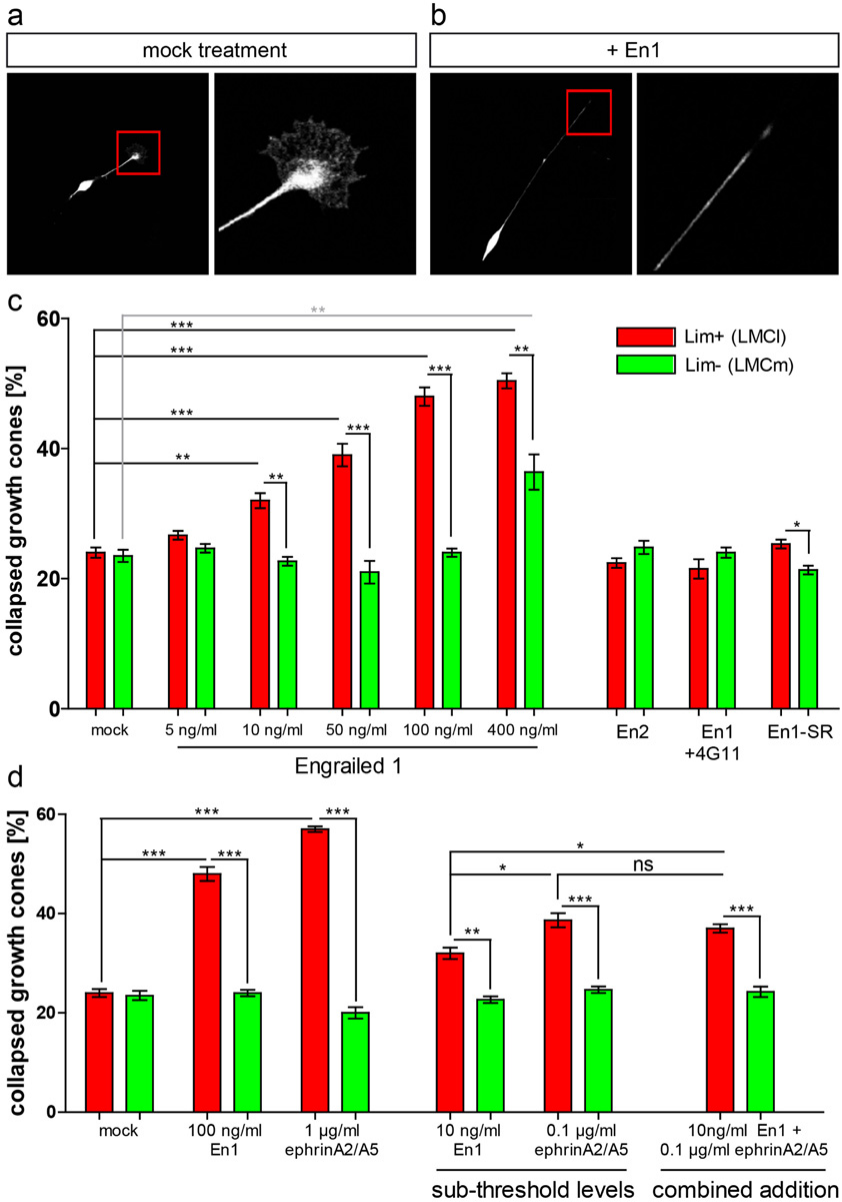
Engrailed 1 specifically collapses lateral LMC growth cones *in vitro* independent of ephrinA5. **(a)** Primary cultured E11.5 motor neurons extend axons and elaborate growth cones after 5 h in culture. **(b)** Addition of En1 protein collapses lateral LMC growth cones in a dose-dependent manner. **(c)** Quantification of growth cone collapse. Immunohistochemical detection of Lim1 was used to discriminate between lateral (Lim1+, red bars) and medial (Lim1-, green bars) LMC neurons, respectively. Pre-incubation of En1 protein with an antibody against En1 (4G11) abolished the collapse-inducing activity of En1. En2 and the internalization-incompetent mutant of En1, En1-SR, did not induce growth cone collapse at any concentration tested. **(d)** Sub-threshold levels of En1 (10ng/ml) and ephrinA2/5 (0.1μg/ml) were determined and added to the motor neuron cultures. The combined addition of En1 and ephrinA2/5 did not result in any additive effects on growth cone collapse.

Next to their well-established intracellular role as transcription factors, Engrailed proteins have been shown to exert an additional, extracellular repellent action on *Xenopus* and chick temporal retinotectal growth cones *in vitro* and *in vivo* [16,17]. To investigate whether En1 has also a direct effect on motor axon growth cones, we cultured primary LMC motor neurons of E11.5 *Hb9*::*eGFP* embryos where green fluorescent protein (GFP) is expressed under the control of the *Hb9* promoter in all motor neurons, thus conveniently labeling motor axons [19]. After 5 hours in culture, either En1 protein was added to the motor neurons or the cultures were mock treated. After fixation, motor neuron cultures were stained for Lim1 to allow for distinction between lateral and medial LMC neurons. Collapsed versus non-collapsed growth cones were scored (Fig. 3a, b) and assigned to columnar identities, i.e. lateral LMC (*Hb9*::*eGFP* ^+^/ Lim1^+^) or medial LMC (*Hb9*::*eGFP* ^+^/ Lim1^-^).

Exposure to recombinant En1 protein specifically induced collapse in lateral LMC growth cones in a dose-dependent manner already at concentrations of 10 ng/ml (corresponds to a 200pM physiological concentration): in cultures treated with En1 protein, 32% ± 1.15 SEM of growth cones from Lim1-expressing motor neurons collapsed, compared to 24% ± 0.82 SEM in mock treated cultures (p<0.01, Fig. 3b, c). With increasing concentrations of En1 in the medium also the percentage of collapsed LMCl growth cones increased to 39% ± 1.73 SEM at 50ng/ml (p<0.001), 48% ± 1.41 SEM at 100ng/ml (p<0.0001) and 50.4% ± 1.17 SEM at 400ng/ml (p<0.0001) when compared to mock treated growth cones (Fig. 3c).

Quite contrary, Lim1-negative medial LMC growth cones were largely resistant to En1 at concentrations from 5ng/ml to 100ng/ml when compared to Lim1-positive neurons (p^10ng^<0.01; p^50ng^<0.001; p^100ng^<0.0001) and showed only a partial collapse response at the highest concentrations tested (4nM, 36.4% ± 2.71 SEM, p<0.01) when compared to mock treated Lim1-negative growth cones (23.5% ± 0.96; Fig. 3c). However, even at the highest concentration of En1 protein added to the cultures, the numbers of observed collapsed growth cones of Lim^-^ LMCm motor neurons were significantly lower than those of Lim^+^ LMCl growth cones (p^400ng^<0.01). Pre-incubation of recombinant En1 with antibodies against En1 (4G11) eliminated the collapse-inducing activity completely (Fig. 3c).

To further investigate the mechanism of growth cone collapse we used an En-1 isoform of which the Penetratin-domain is mutated and therefore is unable to be internalized (En1-SR, [16]). Exposure of cultured motor neurons to En1-SR did not result in growth cone collapse above base line levels of mock treated neurons (25.33% ± 0.67 SEM Lim1+ vs. 24% ± 0.82 SEM mock, Fig. 3c), indicating that En1 activity is dependent on uptake into cells. We next asked whether this collapsing activity is specific to En1 or whether all homeodomain proteins can elicit the same response. We therefore tested a close relative of En1, namely En2 that elicits growth cone turning in *Xenopus* tectal neurons [16] and can substitute lost En1 function in the formation of the mid-hindbrain boundary [18]. However, when applied to primary motor neurons, En2 was not able to induce collapse of growth cones at any concentration used (Fig. 3c).

Thus, En1 protein possesses a strong and specific growth cone collapse-inducing activity upon internalization by dorsally projecting LMCl axons.

### EphrinA5 does not act synergistically on En1-induced LMCl growth cone collapse

In the chick retinotectal system, Engrailed proteins cooperate with ephrinA5 in the anterior-posterior mapping of retinal axons. More specifically, extracellular En1 or En2 increase the sensitivity of retinal growth cones to sub-threshold levels of ephrinA5 in the surrounding environment [17]. In the developing forelimb, ephrinAs that are expressed in the ventral part of the limb have been shown to mediate repulsion of lateral LMC axons through the EphA4 receptor that is expressed on these axons [12,35,36]. We tested whether ephrinA2/5 and En1 also cooperate *in vitro* in mediating collapse of LMC motor axons.

The minimal concentration of ephrinA5 required to elicit repulsion of temporal axons was 0.5μg/ml, while 0.1μg/ml did not repell axons, thus being determined as sub-threshold levels in the tectum [17]. Unlike in the retinal system, addition of 0.1μg/ml ephrinA2/A5 resulted in a significant increase of collapsed Lim1^+^ motor axons (38.67% ± 1.43 SEM) when compared to mock-treated Lim1^+^ motor neurons (p<0.0001) and neurons that were treated with 10ng/ml En1 alone (p<0.02; Fig. 3c, d). Concomitant addition of ephrinA2/5 together with En1 did lead to a slight increase in the number of collapsed LMCl growth cones (37% ± 0.85 SEM) when compared to an exposure to 10 ng/ml En1 alone (32% ± 1.16 SEM, p<0.05; Fig. 3d). However, growth cone collapse of LMCl axons never reached numbers observed upon addition of 1μg/ml ephrins (57% ± 0.58 SEM, p<0.0001; Fig. 3d), and we never observed levels such as the 5-fold increase of collapsed temporal growth cones upon combined addition of ephrins and Engrailed protein [17]. Furthermore, there was no significant difference in the number of collapsed growth cones upon combined addition of ephrins and Engrailed when compared to addition of 0.1μg/ml ephrins alone (p = 0.31; Fig. 3d), thus implicating that addition of ephrins holds responsible for the increase of collapsed LMCl growth cones.

From this we conclude that En1 specifically collapses lateral LMC growth cones and that this function of En1 in the innervation of the limb is not synergistically modulated by interaction with ephrinA2/5.

### Engrailed 1 repels lateral LMC axons in vivo

**Fig 4:**
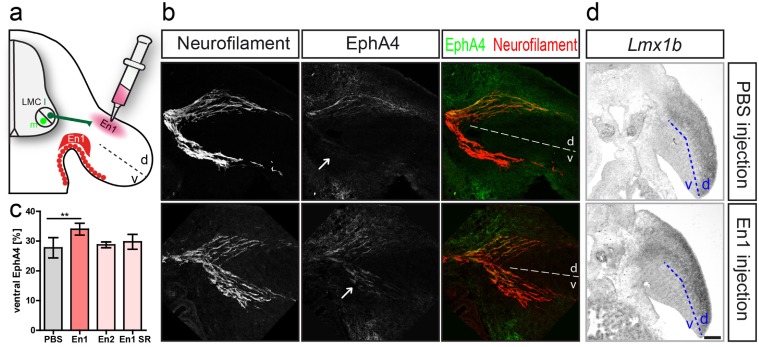
Engrailed controls the dorsal-ventral choice of lateral LMC axons *in vivo*. **(a)** An ectopic source of either En1, En2, or En1-SR was established by injecting the proteins into the dorsal mesenchyme of the developing chick wing. **(b)** Immunohistochemical detection of neurofilament (red) and EphA4 (green) visualizes the dorsal-ventral branching point and EphA4-positive LMCl axons. After injection of En1 into the dorsal wing bud more EphA4-positive axons are present in the ventral branch compared to mock (PBS) injection (arrows). **(c)** Quantification of axonal EphA4 signal results in a higher average EphA4 intensity in the ventral branch after En1 injection when compared to embryos where PBS was injected EphA4 signal was not altered significantly if En2 (28.75% ± 0.48 SEM, n = 4) or En1-SR (29.75% ± 1.25 SEM) were injected into the dorsal wing mesenchyme. **(d)** The expression patterns of the dorsal marker *Lmx1b* were not altered by injection of ectopic En1 into the dorsal wing. Scale bar in d equals 100μm for b and 250μm for d.

In addition to its expression in the ventral ectoderm, En1 is also expressed just ventrally of the dorsal-ventral choice point of the forelimb (Fig. 1c, d), which puts it in a position to exert a repulsive effect on lateral LMC axons also *in vivo*. To investigate whether dorsally projecting motor axons are repelled by En1, we established an ectopic En1-source in the dorsal wing bud of the developing chicken embryo by injecting En1 protein starting at stage 20 to 21 HH until stage 24/25 HH. Evaluation of EphA4 expression on dorsally and ventrally projecting motor branches showed that in embryos where En1 was injected dorsally, a significantly higher amount of EphA4 positive axons was observed on the ventrally projecting branch (34% ± 0.82 SEM, Fig. 4a, b) when compared to embryos that were injected with PBS (27.75% ± 1.7 SEM, p<0.01, Fig. 4c). Interestingly, dorsal injections of either En2 or the uptake-deficient form of En1, En1-SR, did not result in re-routing of EphA4-positive LMCl axons towards the ventral nerve branch. To exclude any patterning effect of the ectopic En1 we monitored the patterning of the wing bud using *Lmx1b* as a dorsal marker. The expression domain remained unchanged when En1 was injected into the dorsal wing compared to PBS injections (Fig. 4d) and we thus conclude that En1 did not act as a ventralizing transcription factor when ectopically injected into the dorsal wing. Taken together, these data show that En1 is a repulsive guidance cue for lateral LMC axons *in vivo*.

## Discussion

### En1 expression in the ventral limb ectoderm controls forelimb patterning

In a typical vertebrate limb, more than 50 muscle groups require precise innervation from corresponding motor neurons in the LMC to establish proper functionality. Secreted or membrane-bound axon guidance molecules that repel or attract advancing growth cones ensure faithful targeting of the respective destination areas. Simultaneously, these target areas need to be appropriately patterned and have to express cues to interact with approaching axonal projections. From E9.5 on, En1 is expressed in ventral limb ectoderm [37], triggering expression of cues relevant in axon guidance, e.g. of the ephrin family, while antagonizing expression of dorsal limb patterning genes, e.g. *Wnt7a* [38]. Considering this, skeletal deficits such as polydactylia or fused bones, as well as normally dorsally positioned nails and hair follicles that are also present along ventral surfaces, and absence of ventral features such as eccrine glands or ventral tendons in *En1* ^*-/-*^ mutants may very well result from ectopic expression of dorsal limb markers in the ventral limb compartment [8,39,40]. However, consequences of these morphological alterations for guidance of motor axons that innervate the developing limb have not been analyzed up to now. Differential expression of homeobox transcription factors confers the ability to motor neurons to select specific axonal pathways: *Lim1* expression, e.g. mediates the expression of the EphA4 receptor on axons of the LMCl and thus repulsive interaction with ephrinAs in the ventral limb, promoting a dorsal trajectory [11,12]. Absence of *En1* expression from spinal motor neurons [41] excludes a direct, cell-autonomous contribution of the transcription factor to dorsal-ventral guidance decisions LMC axons. Upon loss of *En1* expression, levels of *ephrinA5* in the limb mesenchyme were diminished, thus interaction with the corresponding EphA4 receptor may not be possible anymore, and thus lead to aberrantly projecting LMCl axons into ventral limb regions in *En1* ^*-/-*^ mutant embryos. The relatively small percentage of axons that take aberrant decisions at the dorsal-ventral choice point could be explained by the existence of other guidance cues that are able to partially correct the trajectory of motor axons. Furthermore, expression of the dorsal limb markers *Lmx1b* and *Npn-2* does only partially mirror the presence of these markers in the dorsal limb compartment in *En1* ^*-/-*^ mutants. Incomplete dorsalization of the ventral limb is also revealed by the expression of the ventral limb marker *Ebf-2* [26,27] that is greatly reduced though not completely absent in mutant mice. Accordingly, *ephrinA5* expression was mainly absent from the axillary region, while more distally it was still expressed. Furthermore, also EphA4 and EphA7, which are direct target genes of Lmx1b, were not upregulated within regions of ectopic expression of Lmx1b which also argues for an incomplete dorsalization of the ventral limb and against the hypothesis that mesenchymal Eph may misguided axons within the forelimb. Therefore, motor axons might only be partially misled at the bifurcation point within dorsalized limb mesenchyme, while in regions that maintain their ventral characteristics they are still able to navigate correctly. The fact that upon mutation of *Lmx1b* expression of EphA4 is only lost within certain regions of the limb, and not entirely [42], shows that Eph expression only partially depends on Lmx1b, and adds to the hypothesis that next to ephrin-Eph signaling other factors are taking part in dorsal-ventral choice decisions of motor axons in the forelimb.

### En1, but not En2, has a direct repulsive effect on LMC axons

Engrailed proteins are multifaceted transcription factors that execute complex functions: the presence of amino acid domains involved in nuclear export, secretion and internalization prompted investigation of the possibility that Engrailed may have a direct role in intercellular communication. Previous investigations showed that in the CNS most of the En1 protein is located within the nuclei of cells, while in the periphery, e.g. in neural crest associated structures such as the cranial mesenchyme or the mandibular arches, it was found in the cytoplasmic compartment of the cells [43]. This difference in subcellular localization may have functional implications on the role of En1 during nervous system development and the establishment of correct peripheral connectivity. The exact mechanism regulating Engrailed secretion is unknown, although it is clear that it depends upon a conserved 11 amino acids sequence called Δ1, which is a part of the nuclear export signal (NES) [44,45]. Engrailed can be internalized and have direct access to the cell cytoplasm and nucleus without the requirement of the classical endocytosis utilizing the 16 amino acids Penetratin sequence within the homeodomain [46]. Studies in *Xenopus* and chick visual systems revealed that Engrailed protein can also act as a direct axon guidance cue, repelling retinal and tectal axons by triggering local translation of proteins that elicit axonal turning [16,17].

Observation of cytoplasmic En1 protein in an axillary position in the developing murine forelimb prompted the question whether dorsal-ventral axonal trajectory errors as observed in *En1* ^*-/-*^ embryos might not only be a result of a mispatterned limb, but also of the absence of direct axonal repulsion by secreted En1 protein.. Indeed, exposure of primary LMCl motor neurons to En1 protein showed a dose-dependent, specific collapse of growth cones. Furthermore, introduction of an ectopic En1 source into the dorsal chicken limb forced dorsally projecting axons into a ventral trajectory. Differently from the phenotypes observed in *En1* ^*-/-*^ mice, ablation of En2 causes milder defects and only light patterning errors. Replacement of the En1 coding sequence with the En2 sequence in *En1* ^*2ki/2ki*^ mutant mice completely rescues all brain patterning and limb phenotypes, demonstrating that function of En1 and En2 is redundant [18,47,48]. Furthermore, a synergistic interaction of Engrailed and ephrinA5 was observed in the retinotectal system, where extracellular Engrailed increases the sensitivity of retinal growth cones to sub-threshold levels of ephrinA5 in the surrounding environment [17]. In contrast to these data, we found that specifically En1 causes collapse of LMC growth cones, while treatment with or ectopic exposure to En2 showed no effect. The fact that endogenous En2 could not rescue defects observed in *En1* ^*-/-*^ mutants demonstrates that the differences in function during brain and limb patterning may be in the main part due to divergent embryonic expression patterns, rather than to differing biochemical capabilities. However, cell type-dependent differences in the biochemical activities of En1 and En2 or the requirement for additional cofactors cannot be excluded: while addition of ephrinAs did not replicate the synergistic repulsive effect on growth cones as observed in the tectum, other potential co-factors, such as Sema3A, Sema3F, and ephrinB2 need to be taken into account for the formation of molecular mechanisms that differentially regulate the establishment of neuronal connectivity within the motor and the visual system.

### Internalized En1 acts specifically on a subset of LMC neurons

Multiple systems of guidance cues work together to precisely establish stereotypic, highly accurate neuronal circuitries, thus, heterogeneity of axons is a prerequisite to react faithfully to environmental information. Previous analyses of axons from temporal and nasal regions of the *Xenopus* and chick retina showed that these axons were repelled or attracted, respectively, only upon internalization of extracellular Engrailed [16,17]. Furthermore, synergistic Engrailed/ephrinA activity was shown to be involved in stimulation of ATP-synthesis and the consequential activation of the adenosine A1 receptor (A1R), which is present at higher concentrations in temporal than nasal growth cones, enhancing growth cone collapse of this specific axonal subset [49]. Utilizing En1-SR protein that cannot be internalized or pre-incubation of recombinant En1 with antibodies against En1 eliminated the collapse-inducing activity, thus showing that internalization of En1 is necessary to elicit growth cone collapse specifically in LMCl axons, while LMCm axons were not affected. These findings argue for specific, yet to be identified mechanisms in subsets of spinal motor neurons upon encounter of extracellular En1. Data arising from the developing visual cortex show that cellular internalization of homeoprotein family members such as Otx2 is facilitated by binding to specific sugar epitopes [50,51]. Next to Penetratin, En1 might use similar internalization mechanisms during uptake into LMCl neurons. Therefore, analyzes of differentially expressed sugar epitopes and putative En1-binding sites in their sequences, as well as receptor molecules such as A1R that depend on En1 action will be helpful to unravel motor neuron subtype specificity.

Next to Engrailed, other homeodomain transcription factors, such as Otx2 in the visual cortex or Pax6 in the retina possess secretion and internalization sequences that enable intercellular passage and potentially allow for direct, non-cell-autonomous activity [50,52]. Intriguingly, intercellular passage has not only been observed in animal models, some of the mechanisms identified also work for plant homeoproteins [53,54], suggesting that homeoprotein exchange might be one of the first primitive ways for cells to communicate. During optic tectum innervation, Engrailed rapidly triggers local translation of new proteins in growth cones that allow for axon turning by direct interaction with eIF4E [16,55]. In motor neuron cultures it is still unclear whether transcription in the nucleus or local mRNA translation is required for En1-mediated repulsive effects. While cultures need to be exposed to recombinant En1 protein for 90–120 minutes to elicit growth cone collapse, pre-treatment with agents to inhibit translation or transcription produced inconclusive results. In the light of our observations upon ectopic injection of En1 protein into the developing chicken wing bud, it appears unlikely, that expression of the homeodomain transcription factor in spinal interneurons [56] indirectly affects motor axon pathfinding from within the neural tube, but from the periphery. Nevertheless, further analyses are required to decypher the mode of action of En1 protein during repulsion of LMCl motor axons: exposure to fluorescently labeled Engrailed allows for localization-analyses of the internalized protein within axon or cell body. Furthermore, collapse studies of axons that were separated from the cell bodies and exposed to En1 will show whether local translation is involved in the collapse response, or if transcription is activated. Loss of function experiments that uncouple whether transcriptional events or local translation regulated by En1 govern guidance decisions may furthermore include the use of secreted or non-secreted antibodies [17]: Antagonizing function of either translational or transcriptional activity of secreted, internalized En1, or endogenous, transcriptional activity of En1 within the nucleus without hampering the respective other pathway will substantially contribute to the understanding of how the transcription factor contributes to dorsal-ventral guidance fidelity. Additionally, other local cues within the limb and their corresponding axonal receptors need to be taken into account for an enhancement of growth cone collapse.

Taken together, our data indicate that En1 is involved in two distinct molecular mechanisms that regulate the establishment of neuronal connectivity to limb-musculature: on the one hand, indirectly, via patterning of the ventral limb and transcriptional activation of guidance cues, such as ephrins, that are essential for proper steering of limb innervating motor axons. On the other hand, our findings point to a direct action of En1 upon internalization on migrating LMCl growth cones implicating that Engrailed protein directly contributes to guidance accuracy of a subset of limb innervating motor axons.

## Supporting Information

S1 Fig EphA4 and EphA7 are not upregulated in ventral limb mesenchyme upon mutation of En1. **(a)** Expression of EphA4 is mainly found I dorsal limb mesenchyme in wildtype and En1-/- mutant embryos. Arrows point to small sites of ventral expression in the forelimb in both wildtype and mutant embryos. **(b)** EphA7 is expressed in axons targeting the dorsal and ventral limb (neurofilament), as well as in dorsal musculature and certain ventral structures at the plexus region and in the proximal limb (arrows) in wildtype and *En1* ^*-/-*^ mutant embryos. Scale bar equals 100μm for all panels
(EPS)
